# Comprehensive Molecular and Cellular Characterization of Acute Kidney Injury Progression to Renal Fibrosis

**DOI:** 10.3389/fimmu.2021.699192

**Published:** 2021-10-29

**Authors:** Renyan Wu, Jiawei Li, Guowei Tu, Ying Su, Xuepeng Zhang, Zhe Luo, Ruiming Rong, Yi Zhang

**Affiliations:** ^1^ Department of Critical Care Medicine, Zhongshan Hospital, Fudan University, Shanghai, China; ^2^ Shanghai Institute of Immunology, Department of Immunology and Microbiology, Shanghai Jiao Tong University School of Medicine, Shanghai, China; ^3^ Department of Urology, Zhongshan Hospital, Fudan University, Shanghai, China; ^4^ Shanghai Key Laboratory of Organ Transplantation, Shanghai, China; ^5^ Biomedical Research Center, Institute for Clinical Sciences, Zhongshan Hospital, Fudan University, Shanghai, China

**Keywords:** acute kidney injury, cisplatin, genetic landscape, ischemia reperfusion injury, renal fibrosis, T-cell

## Abstract

Acute kidney injury (AKI) and chronic kidney disease (CKD) represent different stages of renal failure; thus, CKD can be regarded as a result of AKI deterioration. Previous studies have demonstrated that immune cell infiltration, oxidative stress, and metabolic mentalism can support renal fibrosis progression in AKI cases. However, the most important triggers and cell types involved in this pathological progression remain unclear. This study was conducted to shed light into the underlying cellular and molecular features of renal fibrosis progression through the analysis of three mouse whole kidney and one human single-cell RNA-sequencing datasets publicly available. According to the different causes of AKI (ischemia reperfusion injury [IRI] or cisplatin), the mouse samples were divided into the CIU [control-IRI-unilateral ureteral obstruction (UUO)] and CCU (control-cisplatin-UUO) groups. Comparisons between groups revealed eight different modules of differentially expressed genes (DEGs). A total of 1,214 genes showed the same expression pattern in both CIU and CCU groups; however, 1,816 and 1,308 genes were expressed specifically in the CCU and CIU groups, respectively. Further assessment of the DEGs according to the Kyoto Encyclopedia of Genes and Genomes (KEGG) enrichment pathway and Gene Ontology (GO) showed that T-cell activation, fatty acid metabolic process, and arachidonic acid metabolism were involved in the fibrosis progression in CIU and CCU. Single-cell RNA-sequencing data along with the collected DEGs information also revealed that the T-cell activation mainly happened in immune cells, whereas the fatty acid metabolic process and arachidonic acid metabolism occurred in tubule cells. Taken together, these findings suggest that the fibrosis process differed between the CIU and CCU stages, in which immune and tubule cells have different functions. These identified cellular and molecular features of the different stages of fibrosis progression may pave the way for exploring novel potential therapeutic strategies in the clinic.

## Introduction

Kidney diseases can be classified into glomerular, tubulointerstitial, and renal vascular diseases based on anatomic features. They can also be divided into acute kidney injury (AKI) and chronic kidney disease (CKD). Ischemia reperfusion injury (IRI) is a pathophysiological phenomenon triggered by the damage caused by reperfusion of ischemic tissue after reestablishing blood supply (1) and is a common cause of AKI. Administration of cisplatin can also lead to AKI, being characterized by a complex pathophysiological profile that has been associated to cellular uptake and efflux, apoptosis, vascular injury, oxidative and endoplasmic reticulum stress, and inflammation (2). CKD is defined as persistent functional or structural abnormalities of the kidney, in which the excretory function is impaired due to loss of functional nephrons. Overall, AKI and CKD represent kidney failure at different time phases. However, currently, no clinically targeted, highly effective, and with low toxicity drug and/or treatment are available for AKI or CKD. Indeed, clinical treatments are very passive, and the current renal replacement therapies are limited. Therefore, early diagnosis, prevention, and treatment of AKI and CKD are prominent problems in acute and chronic renal injuries. Mechanistic research and enhanced treatment plans for these diseases urgently needed.

The etiology of renal fibrosis progression is complicated; nevertheless, injury is believed to be the main cause. Moreover, the AKI-to-CKD transition is a complex process mainly facilitated by maladaptive repair mechanisms. Several studies have suggested that it is related to inflammation in the kidneys, as many immune cells are recruited to injured organs (3). However, engagement of innate immune receptors provides tubular epithelial cells with the metabolic flexibility necessary for their plasticity during injury and repair, which drives tubule metabolism to also be involved in injury and fibrosis progression (4). Studies have also reported that oxidative stress caused by injury can hamper cell repair and promote tissue fibrosis (5). Despite of these knowledge advances, the underlying reasons for AKI deterioration and renal fibrosis progression remain unclear.

In this study, we found that regardless of whether AKI was caused by IRI or cisplatin, T cell activation was mainly related to renal fibrosis progression. Fatty acid metabolic processes mainly occurred in tubule cells, and most related genes were both involved in IRI and cisplatin-induced AKI, but only few genes were enriched in cisplatin-induced AKI deterioration. However, arachidonic acid metabolism was significantly different in AKI deterioration, in which genes were changed distinctly in different AKI modules. Our findings emphasize the differences in the AKI-to-fibrosis process caused by different factors and connect these functions with the cell types in the kidney, which made a deeper analysis and understanding of fibrosis caused by injury.

## Materials and Methods

### Data Source

Three mouse bulk kidney mRNA-sequencing data (GSE10669, GSE145053, and GSE153625) and one human single-cell kidney RNA-sequencing data (GSE131685) were obtained from the National Center of Biotechnology Information (NCBI) Gene Expression Omnibus database (GEO). All mouse data were based on C57BL/6 mice and comprised eight cisplatin samples, four IRI samples, nine unilateral ureteral obstruction (UUO) samples, and 12 control samples. GSE131685 comprised three healthy kidney samples, including 23,366 cells.

### Data Preprocessing

All raw RNA-sequencing data were obtained using the NCBI SRA (Sequence read archive) Toolkit, and all reads were alignted to the GRCm38 reference genome using *hisat2* (version 2.1.0) algorithm. The average overall mapping rate is more than 90%. After the data was sorted using *samtools* (version 1.7), *htseq* (version 0.11.3) algorithm was used to calculated gene counts.

### Identification of Differentially Expressed Genes

Several groups were compared as follows: (i) UUO *vs.* AKI (IRI or cisplatin), (ii) AKI (IRI or cisplatin) *vs.* control. The batch effect between each sample was removed by *limma (version 3.42.2): remove batch effct. Deseq2* (version 1.24.0) was used to normalize the count data and identify DEGs, and Benjamini-Hochberg correction was used to adjust P-value obtained from multiple test. Finally, genes with |log2Fold Change| > 2 and adjusted P-value < 0.0001 were considered as DEGs.

### Gene Enrichment Analysis

Kyoto Encyclopedia of Genes and Genomes (KEGG) pathway and Gene Ontology (GO) enrichment analyses were performed using *ClusterProfiler* (version 3.14.3) from Bioconductor. An adjusted P-value < 0.05 and false discovery rate < 0.02 were set as the cutoff criteria to identify significantly enriched KEGG pathways and GO terms.

### Single-Cell RNA-Sequencing Data Processing and Analysis

The 10X gene matrix data were loaded in Seurat tool and were filtered based on cells with >3,000 features and 50% mitochondria. The remaining cells were reduced in dimension by Principal Component Analysis (PCA) and then clustered using Uniform Manifold Approximation and Projection for Dimension Reduction (UMAP) tool. Cell type classification was performed using markers as previously described. Specific gene sets were packed using the *AddModuleScore* function to calculate the average expression of each gene set per cell type.

### AKI and CKD Mouse Models

C57BL/6 mice (male, 6–8 w, 20–25 g) were purchased from Shanghai SLAC Laboratory Animal Co., Ltd and maintained in the animal facility in Zhongshan Hospital, Fudan University. Kidney IRI, Cisplatin, and Unilateral ureteral obstruction (UUO) model were as our previous study (6, 7).

### PCR

Total RNA was extracted from mouse kidneys with TRIzol (Invitrogen) reagent according to the manufacturer’s instructions. Total RNA (3–5 μg) was transcribed into cDNA by Superscript II reverse transcriptase and random primer oligonucleotides (Vazyme). The gene-specific forward and reverse primers for mouse RAC2 and CBR1 are presented in **Table 1**. qPCR was carried out using the Hieff qPCR SYBR Green Master Mix (yeason).

**Table 1:** Primers used for qPCR.

Genes	Forward Primer	Reverse Primer
GAPDH	5’-GACTTCAACAGCAACTCCCAC-3’	5’-TCCACCACCCTGTTGCTGTA-3’
RAC2	5’- CAACGCCTTTCCCGGAGAGT -3’	5’- TCCGTCTGTGGATAGGAGAGC -3’
CBR1	5’- CAGAGACCCCTGTGTACTTG -3’	5’- CAACTCAGGACAAGGTACAAAATG -3’

## Results

### Dataset Collection and Analysis Workflow

**Figure 1:**
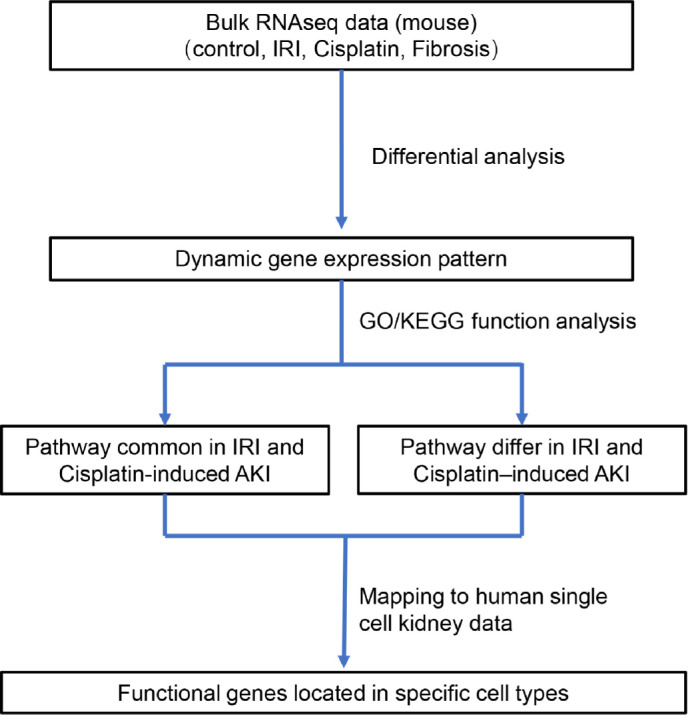
Diagram of the workflow of the study.

Three bulk RNA-sequencing datasets (GSE10669, GSE145053, and GSE153625) comprising 12 AKI samples (including eight cisplatin-induced AKI and four IRI-induced AKI models) and nine renal fibrosis samples (induced by UUO) were evaluated and compared to identify the dynamic gene expression patterns during AKI progression and deterioration. The samples were divided according to the AKI cause in two groups that were used to represent the progression from IRI-induced or cisplatin-induced AKI to renal fibrosis (CIU and CCU groups, respectively). Then, function analysis was performed to identify the common and specific pathways involved in the different AKI-to-fibrosis progression, based on the compared gene modules in CIU and CCU groups. Finally, we mapped the renal fibrosis-related genes to human single-cell data to explore the cell types involved in AKI deterioration and progression (**Figure 1**).

### Gene Expression Analysis and Module Selection

**Figure 2:**
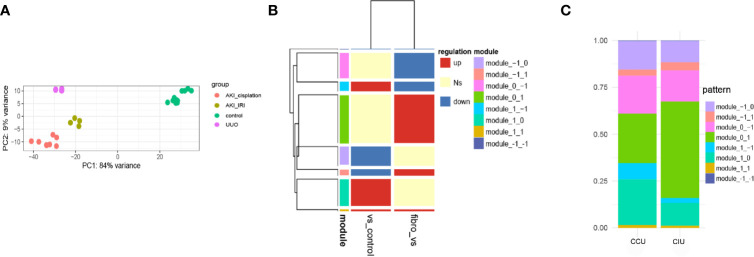
Dynamic gene expression patterns in acute kidney injury (AKI)-to-fibrosis progression. **(A)** Principal component analysis of all datasets. **(B)** Gene expression modules in AKI development and deterioration Fibrosis samples were compared within AKI samples and between AKI and healthy samples. Up shows increased differentially expressed genes (DEGs), down shows decreased DEGs, and Ns shows DEGs that have no significant difference in the comparison. **(C)** The distribution of each module in different AKI progressions. CCU, from healthy to cisplatin-induced AKI to fibrosis; CIU, from healthy to IRI-induced AKI to fibrosis. All data are available at GEO database (GSE10669, GSE145053, GSE153625).

PCA was used to investigate the distribution of the different datasets. The gene expression profiles were found to be significantly different between AKI and renal fibrosis samples; however, IRI-induced and cisplatin-induced AKI groups showed similar gene expression landscapes (**Figure 2A**). Then, we used AKI as a dividing line to separate the progression of fibrosis into early and latter processes. This pattern during the fibrosis process divided the DEGs into eight different modules, and we used −1, 0, and 1 to show the downregulated, no significantly changed, and upregulated gene sets (**Figure 2B**). During the occurrence and deterioration of AKI, the proportion of genes in the different modules changed. Based on the diverse IRI- and cisplatin-induced pathological effects, the module gene ratios during the occurrence and deterioration of the two different AKIs were compared. Overall, a clear difference in the proportion of different gene modules between cisplatin- and IRI-induced AKI was noticeable (**Figure 2C**).

### Common and Specific Functions in CIU and CCU Pattern

**Figure 3:**
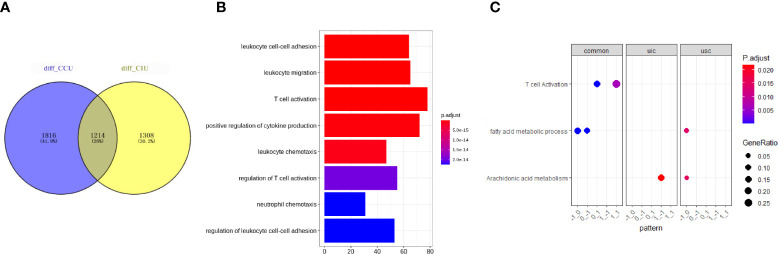
Common and specific functions in CIU and CCU progression patterns. **(A)** Venn plot showing the number of genes in the same or different modules in the CIU and CCU groups. **(B)** Gene ontology enrichment of genes with common expression patterns in CIU and CCU groups. **(C)** Functional analysis of genes in each group. Pattern is referred to as gene modules. CIU (from healthy to IRI-induced AKI to fibrosis) or CCU (from healthy to cisplatin-induced AKI to fibrosis), genes in this pathway are specific to CIU or CCU, respectively; Common, genes in this pathway are shared in the same module of CIU/CCU. The total differential data can be found in **Supplementary CSV File**.

To investigate the commonalities and specificities of the dynamic gene expression patterns during AKI development and deterioration to fibrosis progression, we compared the DEGs identified in the CIU and CCU groups. A total of 1,214 genes were found to have similar expression patterns in both CIU and CCU groups; however, 1,816 and 1,308 genes were expressed specifically in the CCU and CIU groups, respectively (**Figure 3A**). The whole GO analysis of different modules are presented in **Supplementary Figure 1**. Noteworthy, the genes showing the same expression patterns in the CIU and CCU groups were mainly related to T-cell activation and immune cell regulation, such as regulation of T-cell activation and regulation of leukocyte cell-cell adhesion (**Figure 3B**). Furthermore, GO and KEGG analyses confirmed that T-cell activation was enriched in both CIU and CCU exclusive patterns, and genes involved in module_0_1 and module_1_1. Fatty acid metabolic processes were enriched in both CIU and CCU exclusive patterns in module_−1_0 and module_0_−1; however, some genes involved in fatty acid metabolic processes were specifically enriched in the CCU exclusive pattern with module_−1_0. Arachidonic acid metabolism was shared by CIU and CCU exclusive patterns. Some genes were enriched in the module_1_−1 in the CIU exclusive pattern, whereas others were enriched in the module_−1_0 in the CCU exclusive pattern (**Figure 3C**).

### Mapping Common and Specific Genes in Single Cell Data

**Figure 4:**
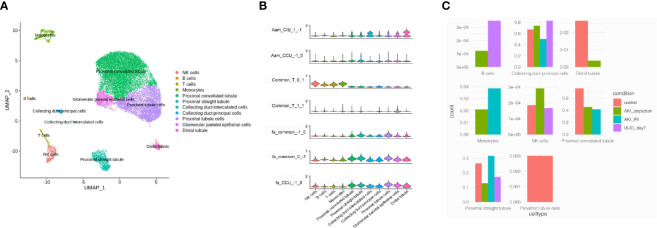
Mapping common and specific genes in single cell data. **(A)** Cell type distribution of human kidney single-cell RNA-sequencing data. **(B)** Enrichment of different pathways in each cell type. Aam, arachidonic acid metabolism; Fa, fatty acid metabolic process; T, T cell activation. CIU (from healthy to IRI-induced AKI to fibrosis) or CCU (from healthy to cisplatin-induced AKI to fibrosis), genes in this pathway are specific to CIU or CCU, respectively; Common, genes in this pathway are shared in the same module of CIU/CCU. 1−1, −10, 01, 11, and 1−1, refer to the gene module. **(C)** Cell counts in different patterns.

To investigate which cell types were involved in the progression of AKI to renal fibrosis, three healthy human single-cell RNA-sequencing data were analyzed to explore the cells that expressed the genes related to renal fibrosis progression. Cells were classified into 11 different cell types, including proximal cells, collecting ducts, distal tubules, natural killer (NK) cells, and T-cells (**Figure 4A**). Then, the functions analyzed from the RNA-sequencing data were merged with the cell types in the kidneys. According to the violin plot, T-cell activation was enriched in the module_0_1, which was identified as a common function in both CIU and CCU patterns, and was mainly related with immune cells, especially in NK cells and T-cells. However, T-cell activation in module_1_1 was mainly mapped to the glomerular parietal epithelial cells. Fatty acid metabolic processes, regardless of the common pattern or CCU exclusive pattern, were found to occur in tubule cells, such as the proximal tubule, distal tubule, and collecting duct. However, arachidonic acid metabolism was different in the CIU and CCU patterns. In the CIU pattern, which was in the module_1_−1, the genes were mainly mapped to the distal tubule and collecting duct intercalated cells, whereas the arachidonic acid metabolism was specifically related to the proximal tubules in the CCU pattern (module_−1_0) (**Figure 4B**). According to the cell change prediction, B cells were increased in the CCU pattern, and the counts of monocyte in the IRI model were higher than them in the Cisplatin-induced AKI model. However, counts of NK cells were increased in the Cisplatin-induced AKI, but decreased when AKI transite to CKD (**Figure 4C**).

### Function of the Identified DEGs in Different Cell Types

**Figure 5:**
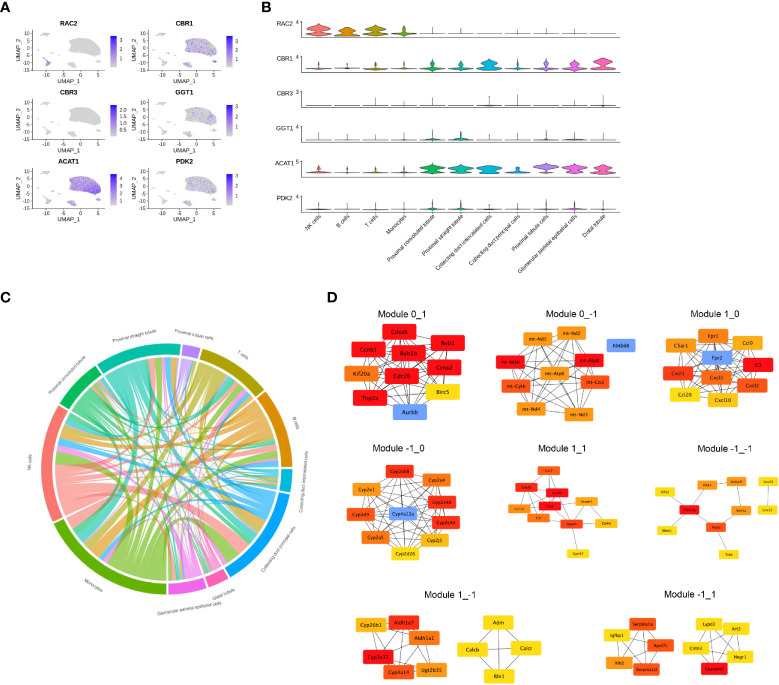
Gene expression profile in each cell type. **(A)** The normalized expression of each gene in the human kidney. **(B)** Violin plot showing the gene expression in each cell type. **(C)** Cell-and-cell interaction between different clusters. **(D)** Protein-and-protein interaction network of different modules.

DEGs identified as having different functions were selected and mapped according to single-cell RNA-sequencing data (GSE131685) (**Figure 5A**). *RAC2* was found to be mainly involved in T-cell activation while enriched in both CIU and CCU groups in module_0_1. Noteworthy, it was mainly expressed in immune cell subsets, especially in NK cells and T cells. Interestingly, *CBR1* and *CBR3* were found to be mainly expressed in tubule cells (comprising collecting duct intercalated cells and distal duct), which were also enriched for arachidonic acid metabolism in the CIU group within the module_1_−1. *GGT1* was also found to be involved in the arachidonic acid metabolism but in the CCU group within the module_−1_0, and it was mainly expressed in the proximal convoluted tubule and proximal straight tubule. *ACAT1* and *PDK2* were identified as being related with the fatty acid metabolic process in the CCU group within the module_−1_0, being expressed in nearly all the tubule cells (**Figure 5B**). Cell-and-cell interactions are presented in **Figure 5C** and protein-and-protein interaction networks of different modules are in **Figure 5D**.

**Figure 6:**
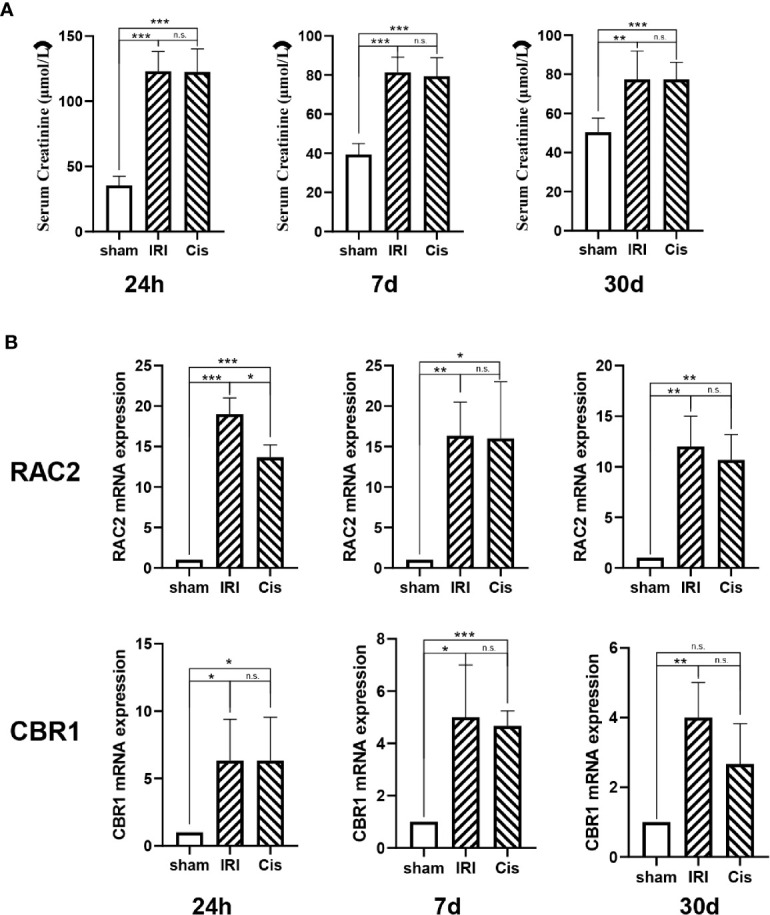
RAC2 and CBR1 mRNA expression in each group. **(A)** Serum Creatinine in sham, IRI, and Cisplatin group after 24 h, 7 d, and 30 d. **(B)** mRNA expression of RAC2 and CBR1 in T cells from kidneys and kidneys. ***P < 0.001, **P < 0.01, *P < 0.05, n.s., no significant differences.

In addition, we established AKI models, RAC2 and CBR1 expression were presented in **Figure 6**.

## Discussion

Progression of the long-term consequences of AKI to renal fibrosis has been well recognized in clinical studies and experimental *in vivo* models. The pathophysiology of AKI relies on a complicated network with multidimensional regulation and the contribution of various factors, such as the immune response, metabolic regulation, and signaling transduction, which in turn results in AKI heterogeneity. IRI- and drug-induced AKI are common in the clinic, both of which contribute to the high incidence of renal fibrosis. Therefore, a better understanding of the cellular and molecular patterns of the progression from IRI- and cisplatin-induced AKI to renal fibrosis may offer promising insights into potential therapeutic advances.

Adaptive immunity, especially T-cell activation, plays an essential role in the progression of AKI. In particular, our previous work showed that inhibition of T-cell immunoglobulin mucin-1-mediated T-cell activation by a histone methylation inhibitor greatly ameliorates renal IRI (6). In a cisplatin-induced AKI mouse model, Miyagi et al. also indicated that T cells with high expression of CD69 and CD25 are present in the spleens of AKI mice after cisplatin treatment (8). Taken together, both studies showed a common pattern of T-cell activation during AKI induction.

The independent characteristics of IRI-induced AKI and cisplatin-induced AKI in renal fibrosis remain unclear. Our analysis indicated that, notably, arachidonic acid metabolism showed completely different patterns during the two processes: the metabolism was enhanced in the phase of IRI-induced AKI, but decreased in the subsequent fibrosis phase, whereas it dramatically decreased in the phase of cisplatin-induced AKI, and remained unchanged thereafter. In a murine model of cisplatin-induced AKI, significant fatty acid oxidation dysfunction and extensive lipid deposition in the mice with AKI were found (9). In addition, histone deacetylase activity (HDAC) was inhibited in kidneys of high-fiber fed mice, and dietary manipulation of the gut microbiome could protect against AKI and subsequent CKD, mediated by HDAC inhibition and activation of two G protein-coupled receptors GPR41 and GPR109A by Short-chain fatty acids (10). Arachidonic acid can be converted into prostaglandin E_2_ by the cascade catalysis of cyclooxygenase (COX)-1 and -2 and prostaglandin E synthase (PGES). Membrane-associated PGES deletion attenuated cisplatin-induced renal dysfunction and tubular damage, suggesting that decreased metabolism of arachidonic acid plays an important role in cisplatin-induced AKI (11). Moreover, Yamamoto et al. also reported decreased arachidonic acid metabolism with the accumulation of COX-2, PGES, and the PGE receptor EP4 in renal tubules (12). In addition, several studies showed that 20-HETE, another important product of arachidonic acid metabolism, is elevated after IRI (13, 14).

Next, the characteristics of common genes correlated with T-cell activation were evaluated. Among these, *RAC2* has been shown to interact with nitric oxide synthase 2A and correlate with renal fibrosis (15). Another study indicated that *RAC2* is a key control gene in the pathogenesis of chronic glomerulonephritis (16). As for the specific genes enriched in different cell types of IRI-induced renal fibrosis, *CBR1* was mainly expressed in tubule cells and some types of immune cells. *CBR1* was reported to be a therapeutic target for hepatic IRI during liver transplantation, as it may be involved in the inhibition of lipid peroxide formation (17). Although no evidence has proven that *CBR1* may function in the process of kidney IRI, Alshogran et al. demonstrated that the expression of *CBR1* and *CBR3* was correlated with end-stage renal disease (18), which highlighted the potential role of *CBR* in IRI-induced renal fibrosis. Based on the analysis of specific genes expressed in cisplatin-induced renal fibrosis, *ACAT1*, *GGT1*, and *PDK2* were predominantly expressed in tubule cells. Indeed, upregulated expression of *ACAT1* was found during renal fibrosis by participating in the oxidation of fatty acids (19). Although no correlation between cisplatin and kidney injury has been shown, several studies have indicated that *PDK2* may mediate cisplatin resistance in head and neck cancer and adenocarcinoma (20, 21). In addition, GGT1 may metabolize cisplatin in the proximal tubule cell, suggesting that the expression of *GGT1* is correlated with the accumulation of cisplatin in the kidneys (22). Taken together, these findings of the characteristics of IRI-or cisplatin-induced AKI may provide information for exploring potential clinical therapeutic targets.

## Data Availability Statement

The raw data supporting the conclusions of this article will be made available by the authors, without undue reservation.

## Ethics Statement

The animal study was reviewed and approved by Zhongshan Hospital, Fudan University.

## Author Contributions

RW and JL analyzed the RNA-sequencing data. GT, XZ and YS drafted the manuscript and established mouse models. ZL, RR and YZ conceived the project, designed the projet, and approved the final manuscript. All authors contributed to the article and approved the submitted version.

## Funding

This study was supported by the National Key R&D Program of China (2018YFA0107501 to RR) and the National Natural Science Foundation of China (81400688 to YZ, and 81770747 and 81970646 to RR).

## Conflict of Interest

The authors declare that the research was conducted in the absence of any commercial or financial relationships that could be construed as a potential conflict of interest.

## Publisher’s Note

All claims expressed in this article are solely those of the authors and do not necessarily represent those of their affiliated organizations, or those of the publisher, the editors and the reviewers. Any product that may be evaluated in this article, or claim that may be made by its manufacturer, is not guaranteed or endorsed by the publisher.

## Supplementary Material

Supplementary Figure 1 GO analysis in each pattern.
