# Supplementary figures and images for: Comprehensive Molecular and Cellular Characterization of Acute Kidney Injury Progression to Renal Fibrosis
Source: Front Immunol. 2021 Oct 29;12:699192. doi: 10.3389/fimmu.2021.699192 (PMC8586649; doi:10.3389/fimmu.2021.699192)

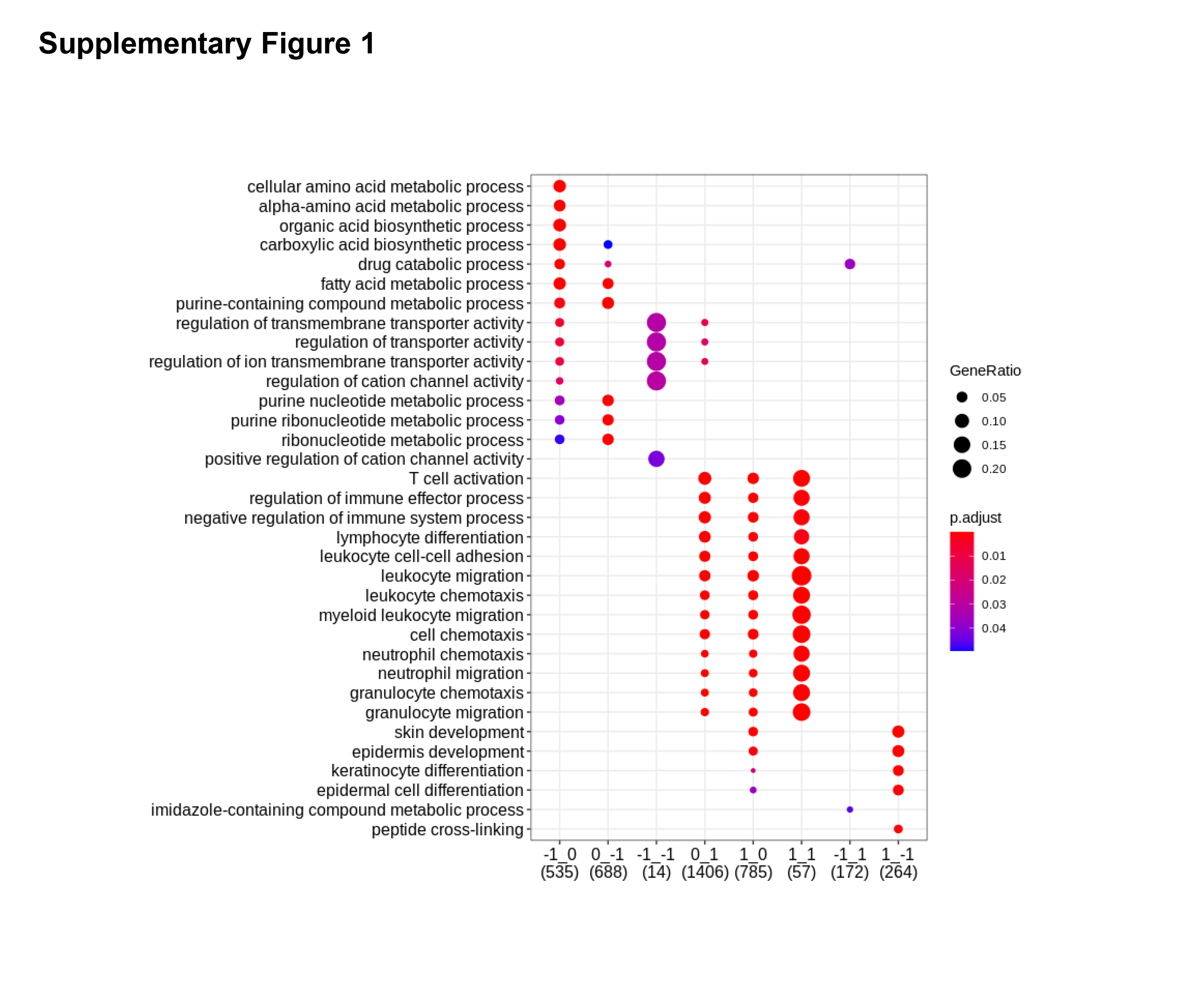

Supplement: Supplementary Figure 1 — GO analysis in each pattern. [file Image_1.tif]
